# Preconception stress exposure from childhood to adolescence and birth outcomes: The impact of stress type, severity and consistency

**DOI:** 10.3389/frph.2022.1007788

**Published:** 2023-01-11

**Authors:** Alison E. Hipwell, Haoyi Fu, Irene Tung, Ashley Stiller, Kate Keenan

**Affiliations:** ^1^ Department of Psychiatry, University of Pittsburgh, Pittsburgh, PA, United States; ^2^ Department of Psychology, University of Pittsburgh, Pittsburgh, PA, United States; ^3^ Department of Biostatistics, University of Pittsburgh, Pittsburgh, PA, United States; ^4^ Department of Psychology, California State University Dominguez Hills, Carson, CA, United States; ^5^ Department of Psychiatry and Behavioral Neuroscience, University of Chicago, Chicago, IL, United States

**Keywords:** preconception, stress exposure, birth outcomes, trajectories, Pittsburgh Girls Study

## Abstract

The negative effects of prenatal stress on offspring health are well established, but there remains little understanding of the influence of stress prior to conception despite known effects on biological systems that are important for a healthy pregnancy. Furthermore, operational definitions of stress vary considerably, and exposure is often characterized *via* summed, ordinal scales of events. We hypothesized that type, severity, and consistency of preconception stress would be associated with birthweight and gestational age (GA) at birth. Data were drawn from a subsample of participants in the 21-year longitudinal Pittsburgh Girls Study (PGS, *N *= 2,450) that has followed women annually since childhood. Prior work in the PGS derived three domains of stress exposure between ages 7-17 years related to subsistence (e.g., resource strain, overcrowding), safety (e.g., community violence, inter-adult aggression), and caregiving (e.g., separation, maternal depression). We tested the effects of dimensions of preconception stress on birthweight and GA among offspring of 490 PGS participants who delivered at age 18 or older (*n* = 490; 76% Black, 20% White, 4% Multiracial). Our hypotheses were partially supported with results varying by stress type and severity and by infant sex. Severity of preconception exposure to subsistence stress was prospectively associated with lower offspring birthweight (*B *= −146.94, *SE *= 69.07, *95% CI *= −282.66, −11.22). The association between severity of caregiving stress in childhood and adolescence and GA at birth was moderated by infant sex (*B *= 0.85, *SE *= .41, *95% CI *= 0.04, 1.66), suggesting greater vulnerability to this type of stress for male compared to female infants. Exposure to safety stressors did not predict birth outcomes. Infants of Black compared with White mothers had lower birthweight in all models regardless of preconception stress type, severity or consistency. However, we observed no moderating effects of race on preconception stress-birth outcome associations. Demonstrating specificity of associations between preconception stress exposure and prenatal health has the potential to inform preventive interventions targeting profiles of exposure to optimize birth outcomes.

## Introduction

Fetal programming provides a model for understanding the development of health and disease that is focused on prenatal conditions that impact the vulnerability of individuals to multiple pathologies (1, 2). Maternal exposure to environmental stressors during the prenatal period is one such condition that has been linked to various suboptimal birth outcomes such as preterm birth and low infant birthweight (3), as well as later neurodevelopmental impairments in childhood (4). The strength of the causal claim that maternal stress has a direct impact on fetal development is based on rigorous controlled experiments in animals, that distinguish prenatal from postpartum effects using methods such as cross-fostering or nursery rearing (5–8).

From a developmental and life-course perspective, however, stress exposure is unlikely to arise *de novo* following conception. In a similar vein, prenatal health is not independent of the health of the system prior to conception. For these reasons, the preconception period is emerging as an important focus for research on adverse birth outcomes and offspring development (9), as well as a model for understanding, and ultimately preventing, health disparities in pregnant women and their children (10, 11).

A small, but growing, evidence base in human studies provides preliminary support for the impact of preconception stress exposure on birth outcomes, although the majority of studies are based on maternal retrospective reports [see review (10)]. For example, at 9-months postpartum, adult participants in the United States’ Early Childhood Longitudinal Study, Birth Cohort (ECLS-B) reported on stressful life events that occurred prior to conception. Results indicated that any exposure to preconception stress was associated with heightened risk for very low infant birthweight, and that the cumulative number of life events was inversely related to infant birthweight (12).

Results of the few prospective longitudinal studies lend some additional support for a link between preconception stress exposure and suboptimal birth outcomes. The National Child Development Study in Great Britain included measures of financial, parenting, family, and community stressors at birth, ages 7, 11 and/or 16 years (13). At ages 33 or 41 years, female participants (*n* ≈ 5,000, 96% White) recalled the outcomes of any pregnancies to date. Results indicated that exposure to stressors across childhood and adolescence was associated with higher rates of preterm birth and lower birthweight, even after accounting for prenatal stress exposure (14). In a follow-up to the National Longitudinal Study of Adolescent Health (Add Health), chronic stressors (e.g., parent receipt of public assistance, high neighborhood unemployment) during adolescence and young adulthood, but not stressful life events were inversely associated with offspring birthweight among the female participants (*n* > 5,000, 57% White, 24% Black, 18% Latina), and also partially explained racial/ethnic disparities in birthweight (15). In the Australian Longitudinal Study of Women's Health, participants' perceptions of stress measured in the three years prior to delivery were linked to state-based data on birthweight for 3,622 women (mostly partnered, highly educated) (16). Results showed no differences in offspring birthweight among women reporting none or minimal stress (survey scores 0 or 1) vs. stress deemed moderate/high. Finally, in a racially diverse sample (44% Black, 30% Latina or Hispanic, 27% White) of low-income women (*n* = 360), earlier GA at delivery was associated with high levels of stress appraisal (i.e., perceived stress and parenting stress) and both high and low levels of exposure to stressors (e.g., life events, financial strain, interpersonal violence) during the interpregnancy interval (17). In general, results of these prospective studies suggest that stress experienced prior to conception can negatively influence birth outcomes, but differences in sample characteristics, conceptualizations of stress and the timing and duration of the exposure window highlight the need for further study.

There are long-standing racial disparities in birth outcomes in the United States with Black women at elevated risk for delivering low birthweight and preterm infants compared to White women (18–20). Increased focus on social determinants of health points to systemic racism and other structural processes as major contributors to these persistent disparities (21–24) relative to individual-level factors (e.g., health behaviors, prenatal characteristics) (25, 26). Despite some evidence that preconception stress explains more variability in birth outcome inequities than stress experienced during pregnancy (27), little is known about the differential impact of type and timing of preconception stress exposure on birth outcomes among Black and White women.

Fetal growth and risk for adverse birth outcomes are known to differ by sex. Whereas birthweights for female infants are generally lower than for males, preterm birth and stillbirths occur more often in male gestations (28–30). Male fetuses may also be particularly susceptible to the negative effects of prenatal stress exposure (31–33), consistent with reports of a decrease in the male-to-female birth ratio in contexts of maternal stress (34–36). However, evidence also suggests that female fetuses show a reduction in growth rate in response to early gestational stress, a response that is considered adaptive (37). Given that most research has focused on stress experienced during pregnancy, the extent to which fetal sex moderates the impact of exposure to *preconception* stress on birth outcomes remains unclear.

The impact of stress exposure on health varies significantly as a function of type, timing, and chronicity, as shown in rodent and non-human primate models (38–42). Results from animal studies also demonstrate that when chronic stress is predictable, rather than unexpected or inconsistent, responses can become habituated and attenuated (43, 44). The study of type, severity and consistency of stress exposure in humans is clearly more complex, given the lack of experimental control and a less developed approach to a functional taxonomy compared to animal studies (45). Extant studies that have measured type and timing of stress in the immediate preconception period suggest that such dimensions explain variance in birth outcomes (14, 15, 17, 46). However, there is a need for longitudinal studies that begin in childhood, assess multiple domains of stress exposure across development, and follow participants through pregnancy and birth to rigorously test the impact of type, severity and consistency of preconception stress exposure on birth outcomes. The Pittsburgh Girls Study (PGS), an ongoing community-based longitudinal study, now in its 21st year of annual data collection, is one such study. Participants (*n* = 2,450) were enrolled at ages 5–8 years and have been interviewed each year about multiple aspects of health and development including exposure to stress. In prior analyses with this sample, we characterized the dynamic nature of three domains stress exposure (subsistence, safety, and caregiving stress) among all PGS participants between ages 7 to 17 years (47). These domains extended from a substantial literature based on animal models (39, 40) and work in humans (48). Analyses indicated variability in initial severity levels, in consistency over time, and timing of shifts in exposure level within- and across-domains. Moreover, group membership differed in terms of racial composition with Black participants over-represented in groups exposed to high and inconsistent levels, especially in the domains of subsistence and safety stress.

In the current study, we examine the influence of these three domains of preconception stress assessed between ages 7 to 17 years on later birth outcomes in PGS participants. We focus on gestational age at birth and infant birthweight given the extensive research on prenatal stress exposure and birth outcomes, the relevance for later health, and clear operational definitions. We hypothesize that high levels of stress exposure (severity) across childhood and adolescence would be inversely associated with gestational age and birthweight. In addition, we expect that changing levels of moderate-high stress (inconsistent exposures over time) would be associated with more adverse birth outcomes given lack of opportunity for adaptation/habituation. We hypothesize that these effects would be evident after controlling for several confounds including maternal age, parity and pre-pregnancy BMI. Finally, we hypothesize that preconception stress effects on birth outcomes would be greater for Black than White women. Because there was insufficient evidence for sex-specific effects of preconception stress exposures, we did not propose hypotheses, but conducted exploratory analyses of potential interactions with infant sex.

## Materials and methods

### Sample

Participants in the PGS were identified in 1999–2000 *via* random household sampling, with over-sampling of households in low resourced neighborhoods. The PGS team enumerated 103,238 Pittsburgh households to locate girls between the ages of 5 and 8 years (49, 50). Neighborhoods in the City of Pittsburgh in which at least 25% of the families were living at or below the poverty level were fully enumerated (i.e., all homes were contacted to determine if the household contained an eligible girl), along with a random selection of 50% of households in all other city neighborhoods. The enumeration identified 3,118 separate households in which an eligible girl resided. From these households, families who moved out of state and families in which the girl would be age-ineligible by the start of the study were excluded. When two age-eligible girls were enumerated in a single household, one girl was randomly selected for participation. Of the 2,992 remaining families, 2,875 (96%) were successfully re-contacted to determine their willingness to participate in the longitudinal study. Of those families, 85% agreed to participate, resulting in a total sample size of 2,450.

As part of the PGS interview starting at age 11, participants were asked annually whether they had become pregnant or given birth in the past year. The present study included participants whose conception occurred at or after age 18 years to establish temporal precedence between stress exposures during childhood and adolescence (up through age 17) and later conception. Participants without birth data from electronic health records were excluded.

A total of 779 PGS participants were identified as having given birth between 2010 and 2021, of whom 59 gave birth before age 18 and had no subsequent births. For participants with multiple births, we focused on the earliest birth with available birth outcome data since the participant turned age 18. Birth outcome data were abstracted from electronic health records for participants who provided consent and delivered at a University of Pittsburgh Medical Center (UPMC) facility (*N* = 429), or for participants who were included in a PGS peripartum substudy and delivered out-of-network (*N* = 61). Among the 720 eligible participants, 490 participants had data for at least one birth outcome (of whom 98.8% had data for both gestational age and birthweight) and infant sex (see flowchart in Supplemental Figure). Thus, subsequent analyses focused on this analytic sample of 490 participants.

Missing birth outcome data were largely due to PGS participants delivering outside the UPMC network. Examination of patterns of missingness revealed that, compared with included participants (*n* = 490), participants without birth data (*n* = 230) were more likely to be living out of state [*Χ*^2^_(1) _= 51.94, *p *< .001], be primiparous [*Χ*^2^_(1) _= 20.76, *p *< .001], identify as White race [*Χ*^2^_(2) _= 30.18, *p *< .001] and to be younger age [*F*(1,718) = 52.89, *p *< .001]. Primary caregivers of excluded participants reported lower levels of perceived stress [*F*(1,718) = 52.89, *p *< .001]. There were no group differences in terms of the proportion of families receiving public assistance.

### Measures

**Birth outcomes.** Gestational age at birth (GA) measured in days and partial weeks and infant birthweight measured in grams were obtained from maternal or child electronic health records.

**Stress domains** were derived in analyses previously described in (47). Preconception stress exposures across three conceptual domains (subsistence, safety and caregiving stress) were obtained from annual PGS interviews with the caregivers when girls were aged between 7 and 17 years. Items within each stress domain are summarized in Table 1.

**Table 1:** Items within each stress domain.

Stress Domain	Subdomain	Items
Subsistence	Resources	• Receipt of public assistance (DEMO) • Trouble with bills/credit rating (DLC) • Long term debt (DLC)
	Housing	• Overcrowding (DEMO) • No suitable/affordable living (DLC) • Trouble with landlord (DLC)
Safety	Neighborhood	• Neighborhood crime (YN) • Witness/victim of a crime (CPC) • Safe on Streets (COMS)
	Domestic	• Abuse of children (DLC) • Abuse of caregiver (DLC) • Inter-adult aggression (CTS)
Caregiving	Disruptions	• Caregiver away from home in past year (DEMO) • Child lived away from home (>1 month) in past year (DEMO) • Change in caregivers (DEMO)
	Strain	• Maternal depression (BDI) • Maternal stress (PSS) • Low warmth (PCRS)

*Subsistence Stress*. Resource-related stress was based on caregivers' reports (yes/no) of receipt of public assistance (e.g., Nutrition Program for Women, Infants, and Children, food stamps, welfare, Medicaid) on the Demographic Questionnaire (DEMO; developed for the PGS), trouble with credit rating, and long-term debts (other than a mortgage) as reported by caregivers (yes/no) on the Difficult Life Circumstances measure (DLC) (51). Housing stress included overcrowding, defined as more than 2 people per bedroom as assessed on the DEMO, and suboptimal housing based maternal report of no suitable or affordable place to live (yes/no) and having trouble with the landlord (yes/no) on the DLC. Binary items measuring resource and housing stress were summed to yield a total score for subsistence stress.

*Safety Stress*. Neighborhood safety was assessed by caregiver report on the extent of illegal activities and neighborhood crime (e.g., vandalism, organized crime, drug-dealing, prostitution) using the Your Neighborhood questionnaire (YN) (52). Participants reported the extent to which 17 items were a problem on 3-point Likert scales. Scores falling in the upper quartile indicated neighborhood safety stress. Caregivers also indicated whether they had witnessed and/or were victimized by violent crime (e.g., homicide, assault, rape) (yes/no) on the Police Contacts measure (PC) (52). Finally, caregivers reported on lack of safety on neighborhood streets on the Community Survey (COMS) (53), defined as endorsing “*disagree*” or “*strongly disagree*” with the statement “*I feel safe on the streets in my neighborhood.”* Domestic safety was assessed using items from the DLC including whether any child was being emotionally, sexually or physically abused by anyone, and whether the caregiver had been physically abused by his/her partner. In addition, caregivers reported on inter-adult aggression on the revised Conflict Tactics Scale (CTS2) (54) with domestic violence coded from items: threatening to hit, throwing objects at the other, or slapping/hitting the other. Binary items measuring neighborhood and domestic safety were summed to yield a total score for safety stress.

*Caregiving Stress.* Disruptions in caregiving were based on reports of child separation/out-of-home care (e.g., foster home, special facility) for more than 1 month within a 12-month period (yes/no) and change in primary caregivers (yes/no) assessed *via* the DEMO measure. Caregiving strain was measured by low maternal warmth using six items from the Parent/Child Relationship Scale (PCRS) (55). Items were summed and the upper quartile defined low maternal warmth. Caregiver report of depression was measured using the Beck Depression Inventory-II (BDI-II) (56); a score ≥ 11 was used to indicate a significant level of depression symptoms. Caregiver stress appraisal was measured with the Perceived Stress Scale (PSS) (57). Fourteen items were rated on 3-point scales (*1 = almost never, 2 = sometimes, 3 = never*), summed, and cut at the upper quartile to index high perceived stress. The six binary items measuring caregiving stress were summed to yield a total score.

In prior analyses (47), we used a group-based trajectory modeling approach to identify the number of groups and shapes of trajectories (e.g., linear, quadratic) within each domain: model fit was compared using the Akaike Information Criterion (AIC) and Bayesian Information Criterion (BIC), with lower AIC and BIC values indicating better fit. The magnitude of decrease in AIC and BIC fit indices with each increase in number of groups was also considered to ensure parsimony. The stress trajectory groups were defined in terms of initial severity level: low (defined as z-scores below −0.5), average (z-scores between −0.5 and 0.5), moderate (z-scores above 0.5 but below 1.0), and high (*z*-scores at or above 1.0). Change in level of exposure over time was defined using a change of greater than 0.5 SD. Thus, consistent exposure was defined by scores that remained within 0.5 SD, and inconsistent (e.g., increasing, decreasing) exposure by changes of at least 0.5 SD.

**Covariates**. Maternal age at conception was estimated by subtracting 40 weeks from the participant's age on the date of delivery. Participants reported on racial identity as part of the PGS. Pre-pregnancy body mass index (i.e., BMI from the PGS wave prior to conception) was calculated from participants' height and weight measured using a stadiometer and digital scale (BMI; kg/m^2^). Infant sex assigned at birth was obtained from medical record and/or maternal report as for the birth outcomes. Given that nulliparity and adolescent childbirth are associated with higher risk of preterm birth and low birthweight (58, 59), we also covaried parity and history of childbirth prior to age 18 using data from annual PGS interviews with measures starting at age 11.

### Procedure

Approval for all study procedures was obtained from the University of Pittsburgh Human Research Protection Office. Written informed consent from the caregiver and verbal assent from the girl were obtained through age 17, whereas all participants aged 18 and older provided written informed consent.

### Data analysis plan

Linear regression models were used to test associations between types of preconception exposure (i.e., subsistence, safety, caregiving stress) and offspring birth outcomes (i.e., GA, birthweight). First, for each of the three stress types, we regressed GA on the stress trajectory groups (Models 1a, 2a, 3a in Tables 4–6 respectively), using the lowest severity group as the reference group. Parallel models were conducted with birthweight as the outcome. Covariates (i.e., maternal age, race, pre-pregnancy BMI, infant sex, birth number, and history of childbirth prior to age 18) were included in the models given their documented associations with birth outcomes. The analytic sample size after adjusting for covariates was *N* = 467. Because there were no differences in GA or birthweight for participants with complete covariates compared to those missing covariates, missing data were handled *via* listwise deletion.

**Figure 1:**
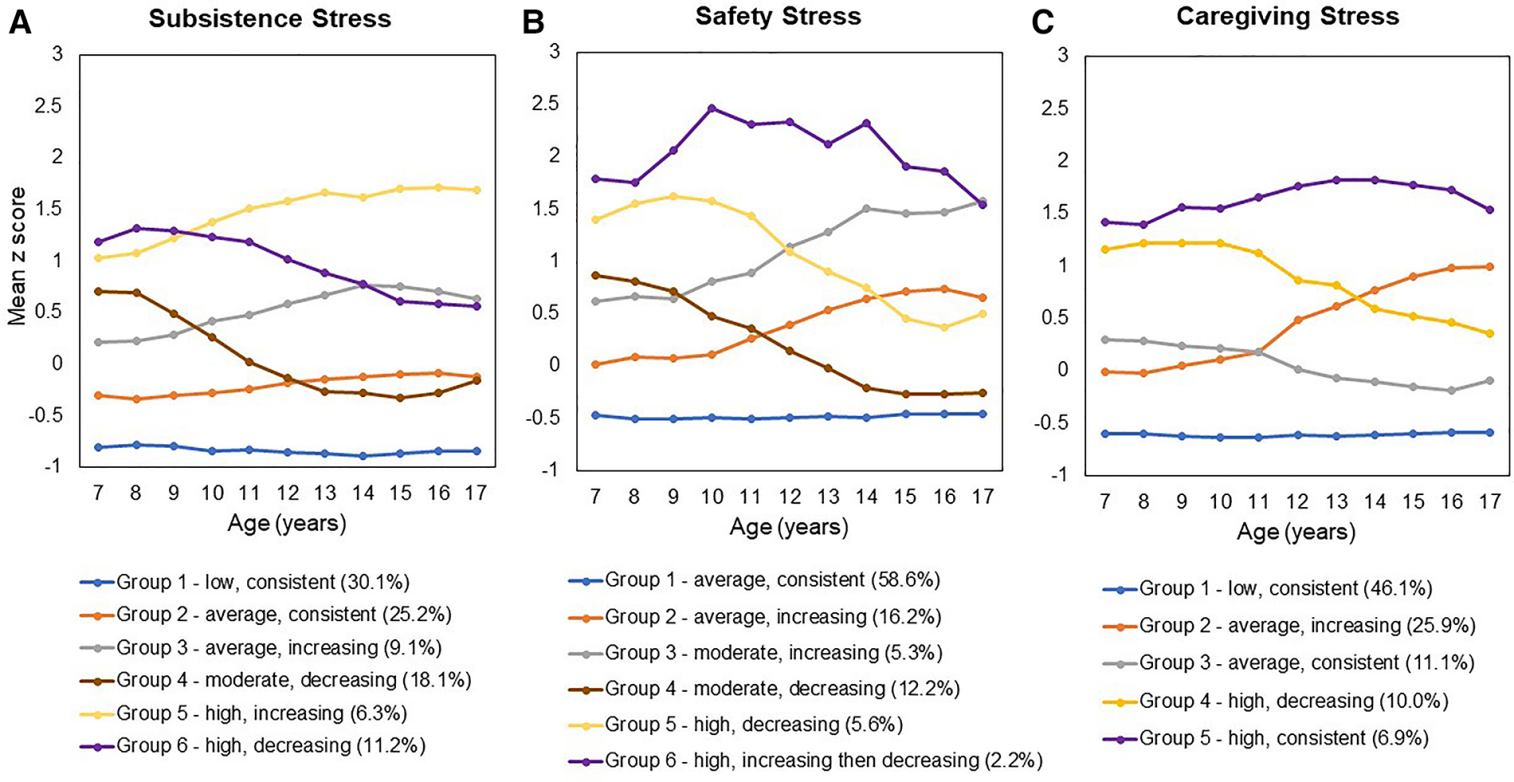
(A) Subsistence Stress, (B) Safety Stress, and (C) Caregiving Stress. [Figure reproduced from the authors' previously published work: Keenan et al. (47)].

As illustrated in Figure 1, the original stress trajectory groups differed from each other with respect to the *consistency* of exposure, as well as *severity* of stress exposure, aspects of stress exposure that have been theorized to uniquely influence birth outcomes (10). In order to increase power, we regrouped the trajectories based on consistency and severity to probe these dimensions of stress exposure. Classification of the original latent stress groups and the binary stress groups used for the present study are shown in Table 2 along with sample sizes for each group. Specifically, to examine differences by *consistency* of exposure, we recoded the trajectory groups into a binary group variable (“stress consistency”) that compared all stress groups following a stable/consistent pattern to all inconsistent stress exposure groups (i.e., increasing and decreasing groups). We then regressed the birth outcomes (GA and birthweight) on the stress consistency group variable for each of the three stress types (Models 1b, 2b, 3b), adjusting for covariates. Next, to examine differences by *severity* of exposure, we recoded the trajectory groups into a binary group variable (“stress severity”) that compared the lowest severity trajectory with all other trajectories. We then regressed the birth outcomes (GA and birthweight) on the stress severity group variable for preconception subsistence and caregiving stress types (Models 1c and 3c) adjusting for covariates. Because safety stress showed only one consistent group, which was also the lowest severity safety stress group, a binary indicator of safety stress severity was not modeled.

**Table 2:** Classification of stress trajectory groups and sample sizes for each domain.

Group categories	Stress domains (*N* = 490)		
	Subsistence stress	Safety stress	Caregiving stress
Original latent groups	Low, consistent (*N* = 91)	Average, consistent (*N* = 217)	
	Average, consistent (*N* = 112)	Average, increasing (*N* = 104)	Low, consistent (*N* = 196)
	Average, increasing (*N* = 63)	Moderate, increasing (*N* = 37)	Average, increasing (*N* = 98)
	Moderate, decreasing (*N* = 107)	Moderate, decreasing (*N* = 84)	Average, consistent (*N* = 84)
	High, increasing (*N* = 52)	High, decreasing (*N* = 33)	High, decreasing (*N* = 61)
	High, decreasing (*N* = 65)	High, increasing, then decreasing (*N* = 15)	High, consistent (*N* = 51)
Stress Consistency	Consistent (*N* = 203)	Consistent (*N* = 217)	Consistent (*N* = 331)
	Inconsistent (*N* = 287)	Inconsistent (*N* = 273)	Inconsistent (*N* = 159)
Stress Severity	Low severity (*N* = 91)	–	Low severity (*N* = 196)
	Average-to-high severity (*N* = 399)	–	Average/high severity (*N* = 294)

Because safety stress showed only one consistent group which was also the lowest severity safety stress group, safety severity was not modeled.

Finally, to examine potential moderation effects by infant sex and maternal race, we conducted follow-up regression models for the stress severity and stress consistency models that included Stress x Infant Sex (male/female) and Stress x Maternal Race (Black/White) interaction terms. Following standard guidelines for interpreting interactions (60), significant Stress x Infant Sex interactions were probed by examining the associations between stress and birth outcomes when the binary sex variable was centered at 0 = male or 1 = female.

## Results

### Preliminary analyses

Descriptive statistics and bivariate correlations among continuous variables are included in Table 3. The sample included 371 Black mothers (76%), 98 White mothers (20%) and 20 Multiracial mothers (4%). One additional participant who identified as Asian American was excluded from the final analytic sample due to the small cell size preventing examination of group differences. Mean age at the time of conception was 22.8 years (SD = 2.8, range = 18–29.6 years). Approximately half (49%) of infants were female. Most of the sample (64.7%) was primiparous, 27.6% had one prior child and 7.7% had more than one prior child. A minority of mothers had had a prior birth before age 18 (15.3%). Gestational age at birth and birthweight were correlated in expected directions. Approximately 14.7% of infants in the current sample had low birthweights (<2,500 grams) and 15.9% of infants were born preterm (gestational age ≤ 37 weeks). Female infants had lower birthweights than male infants [*M *= 3014.91, *SD *= 594.16 *vs. M *= 3123.49, *SD *= 550.39, *F*(1,487) = 4.40, *p* = .036] but there were no sex differences in terms of GA at birth. Infants of Black mothers compared with infants of White mothers had significantly lower birthweights [*M *= 3017.72, *SD *= 582.00 *vs*. *M *= 3252.45, *SD *= 524.27, *F*(1,467) = 13.12, *p* < .001] and shorter gestations [*M *= 38.64, *SD *= 2.27 vs. *M *= 39.18, *SD *= 1.86, *F*(1,461) = 4.66, *p* = .031]. In categorical terms, low birthweight was more than twice as common for infants of Black mothers (17.0%) compared to infants of White mothers (8.2%), and preterm birth was more common for infants of Black mothers (17.2%) compared to infants of White mothers (11.3%).

**Table 3:** Descriptive statistics and bivariate correlations.

Variables	Mean (SD) or N (%)	1	2	3	4
1. Gestational age at birth (weeks)	38.8 (2.2)	–			
2. Birthweight (grams)	3070.3 (575.1)	.663^a^	–		
3. Maternal age at pregnancy (years)	21 (3.1)	.068	.058	–	
4. Pre-pregnancy BMI	28.4 (7.2)	.075	.044	.097^b^	–

Pearson's correlation coefficients.

^a^ *p* < 0.001.

^b^ *p* < 0.05.

### Exposure to preconception subsistence stress and offspring birth outcomes

Results from the models testing preconception exposure to subsistence stress as a predictor of offspring birth outcomes are shown in Table 4. When comparing the original six subsistence stress trajectories, none of the trajectory groups differed from the low-consistent reference group with respect to predicting offspring GA. In addition, most of the groups did not differ from the low-consistent reference group with respect to birthweight, although offspring of the moderate-decreasing group showed significantly lower birthweight than the low-consistent reference group (Table 4 Model 1a).

**Table 4:** Preconception exposure to subsistence stress predicting offspring birth outcomes.

	Gestational Age				Birthweight			
	*B*	*SE*	*CI*	*p*	*B*	*SE*	*CI*	*p*
*Model 1a: Original Stress Groups*								
Subsistence stress trajectory				.315				.371
Average-consistent	0.02	0.32	[−0.60, 0.64]	.948	−141.10	82.12	[−302.48, 20.29]	.086
High-decreasing	−0.34	0.37	[−1.07, 0.39]	.360	−148.21	96.29	[−337.44, 41.02]	.124
Average-increasing	0.16	0.37	[−0.58, 0.89]	.676	−73.82	96.83	[−264.12, 116.48]	.446
Moderate-decreasing	−0.59	0.32	[−1.23, 0.05]	.069	**−166**.**89**	**83**.**89**	**[−331.75**, **−2.04]**	.**047**
High-increasing	−0.42	0.39	[−1.19, 0.35]	.285	−195.68	101.55	[−395.26, 3.89]	.055
Low-consistent (ref)	–	–	–	–	–	–	–	–
Infant sex: female (ref = male)	0.20	0.20	[−0.20, 0.60]	.323	−90.25	52.6	[−193.62, 13.13]	.087
Maternal race: Black (ref = White)	−0.37	0.27	[−0.90, 0.15]	.162	**−217**.**20**	**68**.**83**	**[−352.47**, **−81.93]**	.**002**
Maternal race: Multiracial (ref = White)	−0.17	0.57	[−1.29, 0.95]	.760	−170.17	147.27	[−459.59, 119.24]	.248
*Model 1b: Subsistence Stress Consistency*								
Inconsistent stress^a^	−0.35	0.21	[−0.76, 0.05]	.089	−65.4	53.69	[−170.91, 40.11]	.224
Infant sex: female (ref = male)	0.17	0.2	[−0.23, 0.56]	.403	−90.08	51.86	[−192.00, 11.84]	.083
Maternal race: Black (ref = White)	−0.35	0.26	[−0.87, 0.16]	.180	**−233**.**93**	**67**.**83**	**[−367.22**, **−100.64]**	.**001**
Maternal race: Multiracial (ref = White)	−0.08	0.56	[−1.19, 1.03]	.889	−181.4	145.67	[−467.66, 104.86]	.214
*Model 1c: Subsistence Stress Severity*								
Average-to-high severity^b^	−0.24	0.27	[−0.77, 0.29]	.374	**−146**.**94**	**69**.**07**	**[−282.66**, **−11.21]**	.**034**
Infant sex: female (ref = male)	0.18	0.2	[−0.22, 0.57]	.375	−94.69	51.73	[−196.35, 6.97]	.068
Maternal race: Black (ref = White)	−0.37	0.27	[−0.90, 0.15]	.160	**−214**.**1**	**68**.**48**	**[−348.67**, **−79.52]**	.**002**
Maternal race: Multiracial (ref = White)	−0.11	0.57	[−1.23, 1.00]	.843	−153.2	146	[−440.12, 133.72]	.295

Models included the following additional covariates: maternal age, pre-pregnancy BMI, parity, history of childbirth prior to age 18. Significant associations are bolded for emphasis.

^a^ Reference group is consistent subsistence stress.

^b^ Reference group is low severity subsistence stress, see Table 2.

To examine differences by consistency of exposure, we compared the low and average consistent exposure groups (*N* = 203) with women exposed to inconsistent subsistence stress (*N* = 287). There was a trend for women with inconsistent subsistence stress exposure in childhood to have offspring with earlier gestational age at birth (*B *= −0.35, *SE *= 0.21, *p* = 0.089). The groups did not differ for birthweight (Table 4 Model 1b). Next, we compared differences by low *vs.* average-to-high severity of exposure to subsistence stress. Compared to the low severity group, mothers with a history of exposure to average-to-high levels of subsistence stress had offspring with significantly lower birthweights (Table 4 Model 1c).

Female infants had marginally lower birthweights than male infants in all models, but there were no sex differences in GA. Offspring birthweight (but not GA) was significantly lower for Black women compared to White women in all models, beyond the main effect of subsistence stress severity (Table 4). Finally, when adding in the stress x infant sex and stress x race interaction terms, neither infant sex nor maternal race moderated the associations between subsistence stress and offspring birth outcomes (all *p*'s > .10).

### Exposure to preconception safety stress and offspring birth outcomes

The results of models testing prediction from preconception exposure to safety stress to birth outcomes are shown in Table 5. None of the original safety stress trajectories differed from the low-consistent trajectory reference group with respect to predicting either GA or birthweight (Table 5 Model 2a). Dichotomizing the safety stress groups into consistent vs. inconsistent exposure also did not significantly explain variability in GA or birthweight (Table 5 Model 2b). Furthermore, inconsistent exposure to safety stress across childhood and adolescence did not interact with infant sex or maternal race to predict either birth outcome (all interaction *p*'s > .10).

**Table 5:** Preconception exposure to safety stress predicting offspring birth outcomes.

	Gestational Age				Birthweight			
	*B*	*SE*	*CI*	*p*	*B*	*SE*	*CI*	*p*
*Model 2a: Original Stress Groups*								
Safety stress trajectory				.348				.395
High-decreasing	0.71	0.42	[−0.11, 1.53]	.090	190.29	107.74	[−21.44, 402.01]	.078
Moderate-increasing	−0.35	0.4	[−1.14, 0.43]	.377	−83.03	103.19	[−285.81, 119.75]	.421
Average-increasing	0.28	0.27	[−0.25, 0.80]	.306	37.2	69.51	[−99.41, 173.80]	.593
Moderate-decreasing	0.09	0.28	[−0.46, 0.64]	.751	61.65	72.69	[−81.20, 204.50]	.397
High-increase-decrease	−0.13	0.59	[−1.29, 1.02]	.819	14.58	151.39	[−282.94, 312.10]	.923
Low-consistent (ref)	–	–	–	–	–	–	–	–
Infant sex: female (ref = male)	0.21	0.2	[−0.19, 0.60]	.301	−78.36	52.08	[−180.70, 23.98]	.133
Maternal race: Black (ref = White)	−0.45	0.26	[−0.97, 0.06]	.081	**−251**.**44**	**67**.**23**	**[−383.56, −119.32]**	**<**.**001**
Maternal race: Multiracial (ref = White)	−0.25	0.56	[−1.35, 0.86]	.659	−209.29	145.21	[−494.66, 76.08]	.150
*Model 2b: Safety Stress Consistency*								
Inconsistent stress^a^	0.16	0.2	[−0.24, 0.56]	.429	45.92	52.52	[−57.28, 149.12]	.382
Infant sex: female (ref = male)	0.21	0.2	[−0.19, 0.60]	.308	−82.03	51.88	[−183.98, 19.92]	.115
Maternal race: Black (ref = White)	−0.44	0.26	[−0.95, 0.07]	.090	**−251**.**28**	**66**.**9**	**[−382.75, −119.81]**	**<**.**001**
Maternal race: Multiracial (ref = White)	−0.19	0.56	[−1.29, 0.91]	.733	−202.03	144.74	[−486.46, 82.40]	.163

Models included the following additional covariates: maternal age, pre-pregnancy BMI, parity, history of childbirth prior to age 18. Significant associations are bolded for emphasis.

^a^ Reference group is consistent safety stress, see Table 2.

### Exposure to preconception caregiving stress and offspring birth outcomes

**Figure 2:**
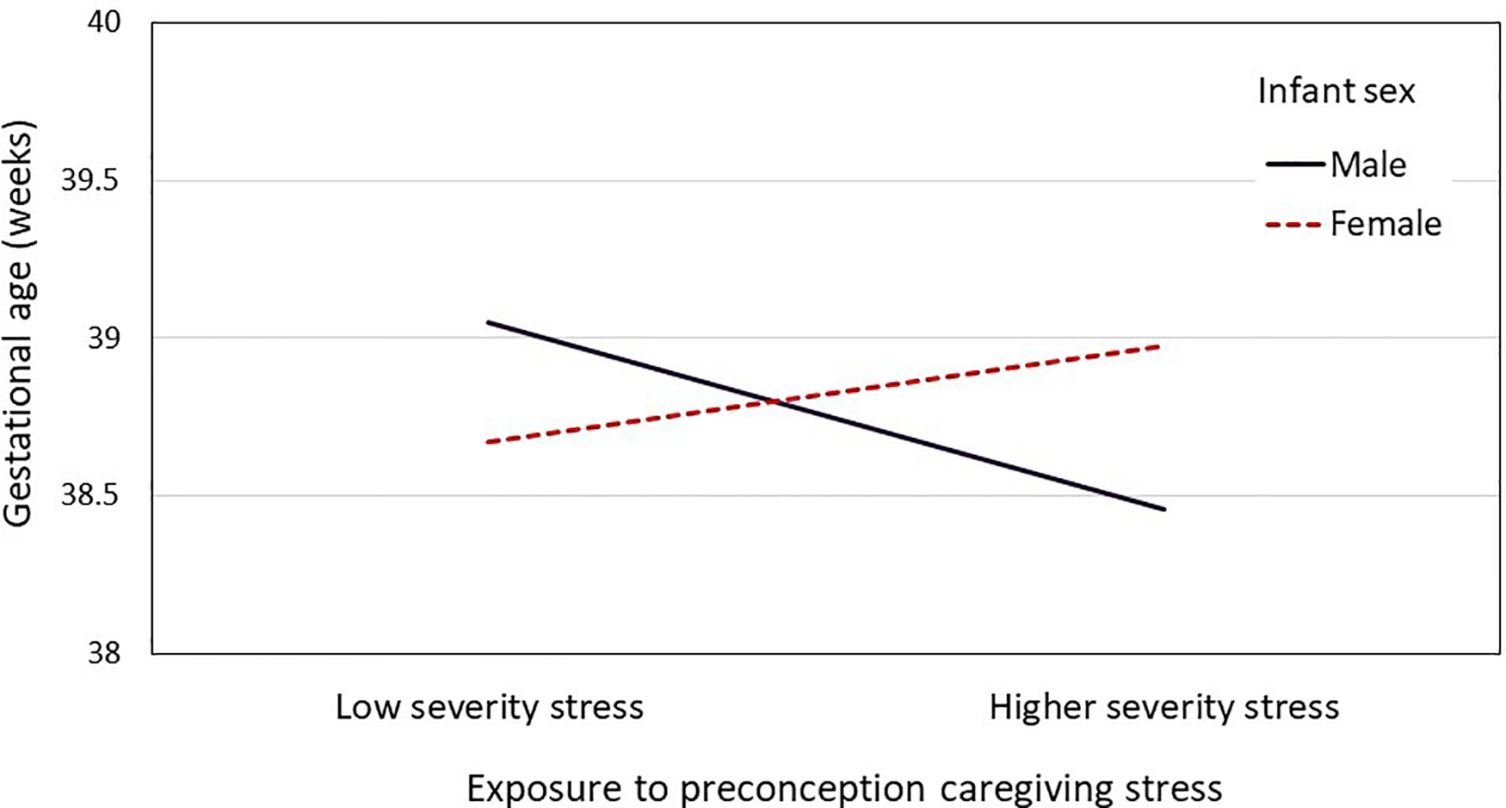
A two-way interaction plot (infant sex and preconception stress severity) for the mean gestational age.

Results from the models testing maternal exposure to preconception caregiving stress in childhood and adolescence predicting offspring birth outcomes are provided in Table 6. None of the original caregiving stress trajectories differed from the low consistent reference group with respect to predicting GA and birthweight (Table 6 Model 3a). Dichotomizing the groups into consistent vs. inconsistent exposure to caregiving stress did not yield significant differences in GA or birthweight (Table 6 Model 3b). Similarly, there was no main effect of average vs. high severity of caregiving stress on birth outcomes (Table 6 Model 3c). However, follow-up analyses showed that the association between severity of preconception exposure to caregiving stress and offspring GA was moderated by infant sex (*B *= 0.85, *SE *= .41, *p *= .039, Cohen's *f* = 0.097). As shown in Figure 2, the pattern of association between severity of caregiving during childhood and adolescence on GA at delivery differed between male infants (*B *= −0.62, *SE *= 0.48, *p *= .198) and female infants (*B *= 0.23, *SE *= 0.51, *p *= .651), although neither simple slope was statistically significant. Infant sex did not moderate the association between severity of caregiving stress and offspring birthweight (*B *= 103.35, *SE *= 105.90, *p *= .330). Finally, maternal race did not interact with severity or consistency of caregiving stress in predicting offspring birth outcomes.

**Table 6:** Preconception exposure to caregiving stress predicting offspring birth outcomes.

	Gestational Age				Birthweight			
	*B*	*SE*	*CI*	*p*	*B*	*SE*	*CI*	*p*
*Model 3a: Original Stress Groups*								
*** ***Caregiving stress trajectory				.269				.584
Average-increasing	0.12	0.28	[−0.43, 0.66]	.675	−87.76	71.43	[−228.13, 52.60]	.220
High-decreasing	−0.54	0.33	[−1.18, 0.10]	.101	−98.04	84.73	[−264.55, 68.48]	.248
Average-consistent	−0.31	0.29	[−0.87, 0.26]	.290	−93.04	74.54	[−239.52, 53.44]	.213
High-consistent	0.28	0.35	[−0.41, 0.97]	.425	50.06	91.01	[−128.80, 228.93]	.583
Low-consistent (ref)	–	–	–	–	–	–	–	–
Infant sex: female (ref = male)	0.16	0.2	[−0.24, 0.55]	.435	−91.81	52.03	[−194.07, 10.44]	.078
Maternal race: Black (ref = White)	−0.37	0.26	[−0.88, 0.14]	.153	**−235**.**66**	**67**.**17**	**[−367.67, −103.65]**	**<**.**001**
Maternal race: Multiracial (ref = White)	−0.18	0.56	[−1.29, 0.92]	.746	−180.18	145.65	[−466.41, 106.04]	.217
*Model 3b: Caregiving Stress Consistency*								
Inconsistent stress^a^	−0.1	0.22	[−0.52, 0.32]	.638	−75.81	55.53	[−184.94, 33.32]	.173
Infant sex: female (ref = male)	0.19	0.2	[−0.20, 0.58]	.344	−88.25	51.74	[−189.92, 13.42]	.089
Maternal race: Black (ref = White)	−0.42	0.26	[−0.93, 0.09]	.104	**−242**.**32**	**66**.**89**	**[−373.77, −110.87]**	**<**.**001**
Maternal race: Multiracial (ref = White)	−0.19	0.56	[−1.29, 0.92]	.739	−197.85	144.6	[−482.02, 86.32]	.172
*Model 3c: Caregiving Stress Severity*								
Average/high severity stress^b^	−0.12	0.21	[−0.52, 0.29]	.575	−68.23	53.11	[−172.60, 36.13	.200
Infant sex: female (ref = male)	0.18	0.20	[−0.21, 0.58]	.360	−90.88	51.89	[−192.85, 11.08]	.081
Maternal race: Black (ref = White)	−0.42	0.26	[−0.93, 0.09]	.104	**−243**.**89**	**66**.**85**	**[−375.27, −112.52]**	**<**.**001**
Maternal race: Multiracial (ref = White)	−0.19	0.56	[−1.29, 0.92]	.740	−198.73	144.63	[−482.95, 85.48]	.170

Models included the following additional covariates: maternal age, pre-pregnancy BMI, parity, history of childbirth prior to age 18. Significant associations are bolded for emphasis.

^a^ Reference group is consistent caregiving stress;.

^b^ Reference group is low severity caregiving stress, see Table 2.

## Discussion

We used multiple waves of repeated data from a longitudinal study to examine the effects of type, timing and consistency of stress exposures during childhood and adolescence on later birth outcomes in a racially and socioeconomically diverse sample of urban-living women. We tested hypotheses that severity and inconsistent exposure to preconception stress would be associated with shorter GA at birth and lower birthweight. Results provided modest support for our hypotheses, showing negative effects of average-to-high levels of preconception subsistence stress (i.e., resource and housing stress) on offspring birthweight. This finding aligns with an established literature describing the pervasive effects of exposure to financial strain in childhood on health across the lifespan (61), as well as results from prior longitudinal and registry studies showing heightened risk for subsequent poor birth outcomes (14, 62, 63). The association is consistent with stress-sensitization and life-course models (64) contending that growing up in a household with income-related physical and social risks may potentiate physiological stress and alter stress regulatory systems (e.g., neuroendocrine, immunological, cardiometabolic) that, in turn, influence prenatal health, placental development and ultimately birth outcomes (65–68). Such a conceptualization integrates early programming (i.e., exposures most impactful during sensitive periods in childhood or adolescence), cumulative exposure (e.g., “wear and tear”) and specificity of type and timing models (10). Early life exposure to subsistence stress may also influence an individual's preparedness for pregnancy *via* long-term deficiencies in nutrition (e.g., dietary fatty acids, vitamin D), infection risk (69, 70), restricted access to quality health care, health screenings, and reproductive health preparations (e.g., folic acid supplementation) (71, 72) and *via* health behaviors such as smoking, disrupted sleep or depression that have also been linked to adverse birth outcomes (73–76). Important next steps will be research focused on subsistence stressors experienced before and *during* pregnancy that may influence fetal growth *via* additive, multiplicative or interactive effects, as well as factors that help explain heterogeneity in birth outcomes despite early adversity.

Stress related to subsistence or financial strain can be relatively stable across the life course (77), and this was evident for approximately 40% of the current sample. Our results, based on annual assessments showed a trend for inconsistent exposure to subsistence stress predicting shorter GA at birth, raising the intriguing possibility that unpredictability is an important feature of subsistence stress that could have implications for prenatal health (10). With a larger sample, future research could probe associations between patterns of inconsistent stress exposure (e.g., early vs. late increasing, childhood increasing and adolescent decreasing trajectories) on birth outcomes, and the extent to which unpredictability in stress exposure continues through pregnancy. In addition, dimensional measures may provide a more sensitive test of the impact of stress related to basic needs across development on perinatal health including birth outcomes.

Our findings also revealed an association between severity of preconception exposure to caregiving stress and GA that was moderated by infant sex. Although the magnitude of the effect was small (Cohen's *f* ≤ 0.25), the direction was consistent with prenatal stress studies that have highlighted male vulnerability (31, 32, 78, 79). Although not examined in the current study, it is possible that prenatal stressors related to early caregiving experiences mediated the effect of preconception stress exposure on birth outcomes. For example, early separation from a parent or exposure to caregiver depression may increase risk for interpersonal difficulties or could activate stressful feelings about parenting that emerge during pregnancy. Mechanistic studies are needed to extend these results and investigate the ways in which stress experienced across the lifespan could alter biological systems that support the healthy development of both male and female fetuses. Improving our understanding of potential selection effects of preconception stress on pregnancy status, pregnancy health and fetal outcomes are also important areas that warrant further research.

Our results showed further indication that the *type* of preconception stress has relevance for birth outcomes. We observed no effects of preconception exposure to neighborhood and domestic safety-related stress on birth outcomes. This result differs from studies documenting associations between exposure to violence in the prenatal period and risk for low birthweight and preterm birth (80) as well as retrospectively reported associations between preconception exposure to trauma (i.e., adult abuse and child maltreatment) on later birth outcomes (81). However, the literature on neighborhood safety is somewhat mixed and small but significant associations are most often reported in studies linking geocoded birth and police-recorded crime data [e.g. (82–84),]. In our prospective cohort study, it is possible that caregiver experiences of safety stress differed from those of the developing child. For example, some caregivers who perceive safety threats engage in higher levels of protective parenting behaviors, which appear to attenuate the impact of the exposure on child health and development [see (85) for a review]. Our dataset includes multiple measures of potential resilience factors (e.g., family support) that will be important for identifying the family and community contexts that mitigate the risks from stress exposure on birth outcomes.

Taken together, the observed patterns of association between type of preconception stress and birth outcomes suggest that there may be differential effects on health systems (e.g., maternal nutrition, endocrine, cardiovascular, or immune functioning) and/or epigenetic modification that warrants further investigation. In prior work with a different sample, we have shown that type of prenatal stress exposure is differentially related to neuroendocrine and cardiac response to a controlled stressor (86), and this may also be the case for preconception stress types. Increasing clarity in operational definitions of stress and a greater focus on the preconception period are critical steps towards filling these gaps and yielding precise, developmentally specific targets for effective preventive interventions.

Birthweight was lower in infants of Black compared with White mothers as has been observed in prior research (21, 22); in the current study, this effect was evident while also accounting for the type, severity and consistency of preconception stress exposures during childhood and adolescence. We previously documented racial differences in stress exposure trajectories based on the full PGS sample (47), and it is also widely recognized that cumulative wear and tear (e.g., weathering, accelerated aging) associated with systemic racism and other structural processes contributes to inequities in Black women's reproductive outcomes (87, 88). In the present PGS subsample of young women giving birth, differences in severity and consistency of stress exposures measured between ages 7–17 years did not account for the risk for low birthweight. Several explanations are possible. First, it is unlikely that our measures of stress, examined as categorical variables in separate models, fully captured the pervasive and multifaceted racial inequalities and lifelong exposure to discrimination experienced by Black women. Second, there may have been important unmeasured influences that occurred before age 7 (e.g., fetal programming, early trauma) and/or after age 17 (e.g., work, education, relationship stress, pregnancy complications and pregnancy-related stress, lack of access to quality prenatal care) that contributed to birth outcomes. Third, it is possible that specific developmental features of childhood-adolescent stress exposures (e.g., moderate or high increasing trajectories) that were associated with observed race differences in the full PGS could not be detected in the smaller childbearing subsample due to reduced statistical power. Large-scale studies with culturally sensitive, multi-level measures of stress and systemic racism across the lifespan are clearly warranted to understand the preconception and prenatal mechanisms that underlie these persistent racial disparities.

## Limitations

Despite some unique strengths of the current longitudinal study, several limitations should also be noted. First, we focused on stress exposures during the formative developmental periods of childhood and adolescence but did not model exposures through to the time of conception or through pregnancy. For example, it is possible that stress type, or changes in severity or consistency in the immediate preconception period contributed to the causal pathway. Results from European population-based registry studies suggest that exposure to stressful life events (i.e., death or serious illness in a close relative) in the 6- to 18-months prior to pregnancy may carry especially high risk as far as preterm birth and low infant birthweight are concerned (46, 89, 90). Understanding the salience and influence of stressors experienced during both the preconception and prenatal periods is critical for informing optimal timing of preventive interventions. Our findings contribute to this effort in demonstrating that, although effects were generally modest, stress exposures in childhood and adolescence may have implications for later birth outcomes and suggest potential benefits of preventive interventions even during the school years. Second, pre-pregnancy BMI was included as a proxy for overall health, but we recognize that BMI is a non-specific measure of perinatal health, especially for Black women (91). Moreover, chronic health conditions (e.g., hypertension or diabetes) that are known risk factors for low birthweight and GA (92) were not included. Third, several factors may have impacted the generalizability of our findings. For example, we focused on births occurring after age 18 to retain temporality between the independent and dependent variables. In doing so, however, we may have excluded births that occurred following especially high levels of preconception stress exposure (93), which probably also reduced our model estimates. Similarly, our focus on live births may have introduced sample bias, including potential bias related to infant sex given that male fetuses are often more vulnerable to loss. In addition, patterns of missing birth outcome data suggested that our results may be most relevant to Black or multi-racial, multiparous, women living in the Pittsburgh region. Fourth, the sizeable, but nonsignificant, birthweight coefficients suggest that the study may have been underpowered to detect causal effects. Finally, while the trajectory modeling approach used in the current study provided new information about severity and consistency of exposures across childhood and adolescence, applying the trajectories to the smaller PGS subsample of pregnant women necessitated reduction into relatively small binary groups that also prevented us from pinpointing developmental periods of heightened plasticity or vulnerability. For example, animal studies have shown that in the peripubertal period, plasticity in the hypothalamic-pituitary-adrenal (HPA) axis increases (94, 95), the effects of which may persist into adulthood (96). Human studies have also shown unique responses to stress around the time of puberty (97).

## Conclusions

The current study supports a life-course perspective for understanding the impact of stress on women's reproductive health with relevance for the next generation. As in other areas of health such as cardiovascular health (98–100), testing the effects of the type, severity and consistency of stress across childhood and adolescence will help to incorporate life history in our understanding and prevention of health problems in pregnancy and the neonatal period. Such an approach is consistent with decade-long calls for the need for preconception physical health care [e.g. (101, 102)], in light of the often modest successes of pregnancy-specific behavioral health interventions (103, 104).

## Data Availability

The raw data supporting the conclusions of this article will be made available by the authors, without undue reservation.
