# Supplementary figures and images for: Preconception stress exposure from childhood to adolescence and birth outcomes: The impact of stress type, severity and consistency
Source: Front Reprod Health. 2023 Jan 11;4:1007788. doi: 10.3389/frph.2022.1007788 (PMC9876597; doi:10.3389/frph.2022.1007788)

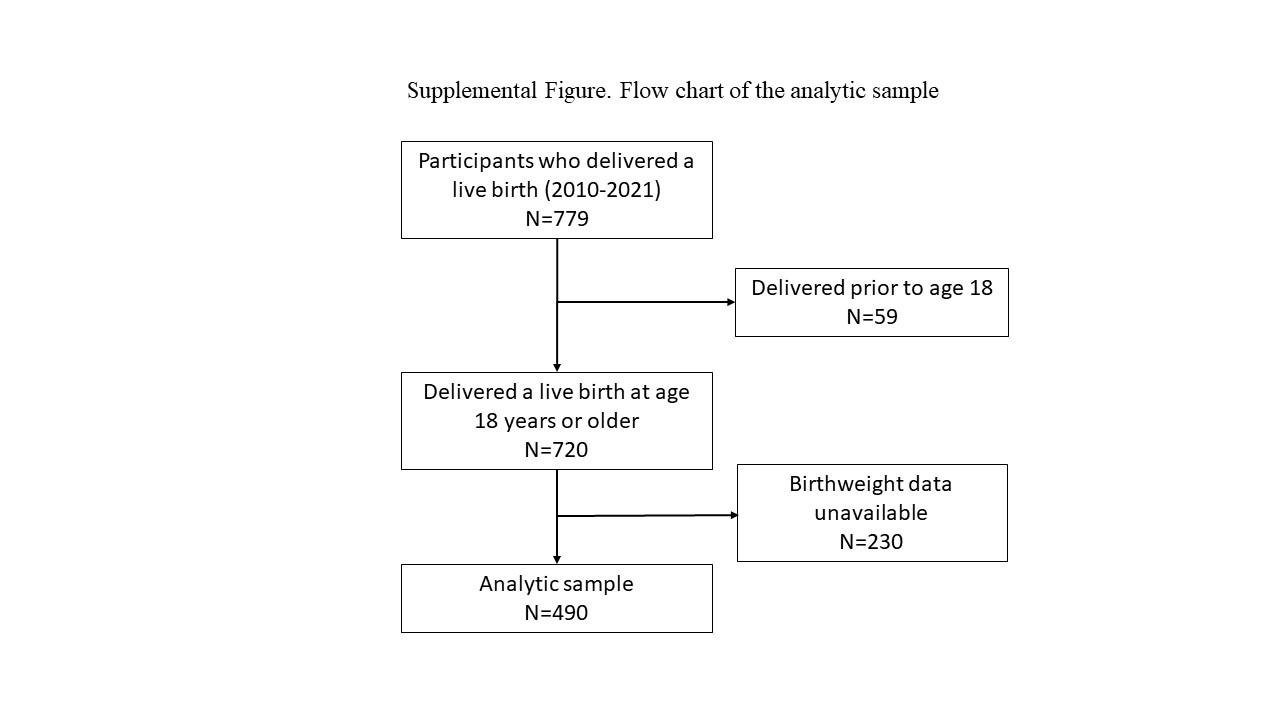

Supplement: Supplementary file 1 [file Image1.jpeg]
